# Stakeholders’ engagement with Ebola therapy research in resource limited settings

**DOI:** 10.1186/s12879-015-0950-8

**Published:** 2015-06-26

**Authors:** Morenike Oluwatoyin Folayan, Brandon Brown, Bridget Haire, Aminu Yakubu, Kristin Peterson, Jemee Tegli

**Affiliations:** Institute of Public Health and Department of Child Dental Health, Obafemi Awolowo University, Ile-Ife, Nigeria; Center for Healthy Communities Division of Clinical Sciences, UCR School of Medicine, 900 University Ave., Riverside, ᅟ, CA 92521 USA; Kirby Institute, UNSW, Sydney, Australia; National Health Research Ethics Committee, Federal Ministry of Health, Federal Secretariat, Abuja, Nigeria; Anthropology Department, University of California, Irvine, CA USA; UL-PIRE Africa Center, University of Liberia, Monrovia, 100010 Liberia

**Keywords:** Ebola, Government, IRB, Communities, Engagement, Research

## Abstract

**Background:**

The current Ebola Virus Disease (EVD) outbreak in West Africa is the largest in history. As of February 18^th^ 2015, 23,258 cases of EVD have been cumulatively reported from Nigeria, Senegal, Guinea, Liberia, Mali, Sierra Leone, Spain, the United Kingdom and the United States of America resulting in more than 9,000 deaths. It is therefore exigent to develop prevention and treatment therapies for EVD.

**Discussion:**

Several new EVD treatments are in clinical development at this time. Based on lessons learned, four critical processes need to be implemented before clinical trials begin. First, all global EVD research need to be coordinated to promote data sharing and synergistic overlap, while reducing unnecessary duplication of efforts. The World Health Organization is well-placed to undertake such an endeavor. Second, governments of affected nations where trials are being proposed need to lead discussions regarding immediate access to any proven medications for epidemics. Also, governments need to leverage international resources to support and expand existing national expertise to jointly conduct high-caliber clinical research; and resources must be used to enhance local technical skills and expand existing personnel. Third, ethics committees must review protocols, monitor the research process, and work closely with research scientists to insure the ethical integrity of research throughout the trials. Fourth, community advisory boards (CAB) need to be formed, linked with existing community leadership structures and organized in conjunction with trial implementation. These community structures should work together with ethics committees to facilitate the study design, informed consent process, and study implementation.

**Summary:**

We must facilitate communication and mutual understanding between trial communities and research teams, and promote positive collaborations between all stakeholders engaged in EVD research. The community engagement process for EVD research is crucial to address myths and misconceptions, and to promote study volunteers’ understanding of the research details. The collaboration between all stakeholders is crucial for continued long term partnership to address EVD outbreak and none of the stakeholders should be left behind in ongoing efforts to develop EVD therapies.

## Background

The current Ebola Virus Disease (EVD) outbreak in West Africa has stimulated renewed interest in the development of treatment and vaccines. As of February 18^th^ 2015, 23,258 cases of EVD have been reported in Nigeria, Senegal, Guinea, Liberia, Mali, Sierra Leone, Spain, the United Kingdom and the United States of America, with a total of 9,380 deaths [1]. Accordingly there is a sense of urgency to develop therapies to curtail the epidemic. Four critical processes need to be implemented for EVD clinical trials in affected countries: 1) government collaboration in countries that host EVD trials with study sponsors to ensure future access to developed products; 2) collaboration between local researchers and their Northern counterparts to ensure transfer of research capacity; 3) development of competent local ethics committees; and 4) empowerment of community members to actively engage in research design and implementation.

Stakeholder engagement in research helps to ensure prompt translation of research findings into policies and programmes. However, unlike most contemporary health research, EVD drug discovery needs to be conducted while the epidemic is ongoing. This brings a sense of urgency for EVD research protocol approval and study implementation, which may increase the likelihood of neglecting stakeholder engagement. This article highlights the merits of creating significant institutional relationships with the four key stakeholders – World Health Organization, national governments, researchers and community members - critical to the conduct of EVD trials, and discusses mechanisms to facilitate these engagement processes.

## Discussion

### Coordination of the global Ebola response

There are several lessons learned about stakeholder engagement in clinical trials from the HIV field. First, all clinical research needs to be coordinated to reduce duplication of effort, promote data sharing, and facilitate research synergy. Despite the criticism that the World Health Organization (WHO) was slow to recognise and respond to the EVD outbreak in West Africa, the agency remains well placed to provide this pivotal coordinating function as an established and acceptable international authority in the region [2]. WHO can facilitate global communication with research consortiums working on EVD research in the region in order to avoid duplication of research efforts, while ensuring rapid information sharing and evaluation of research results.

WHO has an Advisory Group on EVD that provides ongoing independent guidance on the strategic response for outbreaks, with multiple technical guidelines on prevention, treatment and the public health response [3]. WHO has also conducted trainings in collaboration with national and international partners through the support of the United Nations’ Mission for Ebola Emergency Response, in order to achieve the goal of getting 70% of cases isolated and treated, and 70% of the deceased safely buried within 60 days [3]. In addition, they have provided technical support for EVD vaccine access and financing and accelerated development and deployment of rapid, easy-to-use diagnostic tools for EVD [3].

One critical role WHO needs to play in the future is to be stronger and more proactive in its support for national governments of countries that report EVD outbreaks. While the current West Africa epidemic has been acknowledged as unprecedented in magnitude and scope [4], past epidemics also claimed lives [5] and had less international attention. It is important to maintain current EVD surveillance so that both infection and case fatality rates are reduced in future outbreaks in Africa and in other parts of the world. WHO also needs to work in collaboration with the governments of affected nations to ensure prompt access to EVD therapeutic and preventive medications when needed in future epidemics.

### Responsibility of the governments of EVD affected nations

The active engagement of national governments of African nations is required to promote ownership of the response. Their actions could also provide the incentive to invest in the research process, which would bring with it capacity development opportunities for local researchers. Unfortunately, the level of government investment in research in these countries is low, as is the number of researchers who can successfully design and implement clinical trials or access research funding [6]. Governments need to support the development of indigenous competence in order to address the shortage of local expertise. Specifically there is a need to increase local researchers’ ability to conduct cutting-edge research, including clinical trials, leveraging international resources available for current EVD research. Funding should be dedicated to developing a critical mass of skilled researchers who could lead locally relevant health research.

Considering that research regulation is in its infancy in the region [7], support for the development and strengthening of institutions, systems, and mechanisms that would engender robust research regulation regimes in affected countries is essential. Research regulatory policies help promote ethical research conduct. The benefits of developing necessary research regulation infrastructure can be sustained long after the current epidemic. For many countries, it is not the enactment of policies and legislations that is the challenge, but their implementation. Research ethics committees may face challenges monitoring research, even when mandated by government [8]. Yet ensuring local oversight of EVD research is essential to fostering trust in the EVD research enterprise. Multiple approaches for conducting research monitoring should be explored [9]. Mechanisms instituted through the development of local research monitoring competence would be an enduring legacy for each country.

The decision to engage Institutional Ethics Review Committees or National Ethics Review Committees to review EVD related research protocols must be determined by the national policies and guidelines that govern research. Frequently, research projects that are of profound national interest are reviewed by National Ethics Review Committees rather than Institutional Ethics Review Committees. The tension and ensuing controversies between the National Ethics Committee and an Institutional Review Ethics Committee in Malawi over protocol determination for an antiretroviral based research project is an example of why guidelines need to be clear [10]. Therefore, the regulatory research structures in EVD affected countries must be reviewed and where needed, guidelines should be revised regarding how to handle EVD research protocols. Systems for interactions and engagement between National Ethics Review Committees and Institutional Ethics Review Committees also need to be defined where these do not exist.

Active engagement of governments would also enable governments of low-income countries to engage and promote early discussions regarding immediate and affordable access to any proven medications for future epidemics through effective mechanisms. It is essential that any effective medications developed are made available through tiered pricing systems accessible by low income countries and delivered through health systems that are strengthened to facilitate distribution [11]. However, since tiered pricing systems have their own operational challenges, national governments should also try negotiating to obtain the drugs for free or at cost if possible.

In essence, governments need to plan for catastrophic infectious disease outbreaks as they would prepare for disaster management, which entails developing systems of surveillance, environmental management, capacity to manage humane quarantine, and provision of medical services for the sick. International non-governmental organizations (NGOs) need to work with governments to build local capacity in each of these areas.

Foreign governments and donors also have responsibilities. Rid and Emmanuel [12] discussed the ethical imperative for high-income countries to support mechanisms for ensuring prompt future access to medication(s) for low-income countries. Studies on the prevention of HIV, tuberculosis and malaria in Africa and Asia have been heavily supported by developed countries. We have also observed the same for a few other tropical diseases like the eradication of Guinea worm infection in Nigeria and the improvement in maternal and child health in Africa. Similar support and outcomes would be expected for EVD.

While health system strengthening may be a primary need for countries currently experiencing EVD outbreaks, it is important to start addressing how high-income countries can support future access to Ebola medication(s) through fair policies regarding the trade of essential pharmaceuticals. The engagement and commitment of Global Access to Vaccines Initiative (GAVI) to facilitate future EVD vaccine access in affected countries [13, 14] is commendable. Still, there is a need to further explore mechanisms that would promote long term independent access of resource-poor countries to EVD vaccines, therapies and diagnostics. This would require that WHO broker the process of having researchers, drug developers and national governments institute legally binding contracts before the trials commence, to stipulate how nations would have access to the developed vaccines and medications.

### Responsibilities of African researchers

As the competency to conduct clinical trials in many African countries is currently low, African scientists within the continent and in the diaspora need to do more to build research competency in the region. This need in developing countries has dominated many discussions and initiatives of North–South collaborations. African researchers who have been and continue to be part of such research capacity strengthening initiatives need to actively engage in the current effort to conduct basic science, clinical, epidemiological, social science and operations research about EVD prevention, management and control in the region. Efforts should be made to foster more North–South and South-South collaborations for this purpose.

### Community engagement and EVD research

Active community engagement is required as a strategy to foster collaborative partnership and social value of the study for the participant communities [15]. However, the notion of ‘community’ has often been too loosely applied with the risk that those eventually engaged for study purposes are not true representatives of the research participants and their communities. To ensure that community collaboration processes are effective, we propose the adoption of guidelines developed to facilitate meaningful community engagement during HIV prevention research [16–18]. This involves actively building relationship between researchers, communities and other stakeholders, and developing mechanisms that enable community members to have real collaborative decision-making power in the research process. The active engagement of community members in study design and implementation prevents misunderstanding of the goals and processes of research, and helps strengthen science through improving acceptability and understanding of the research, improving the ethics of international collaborative research, countering misconceptions, and increasing familiarity with and trust in trial staff [19]. Community engagement during EVD research especially in the time of an epidemic can address myths and misconceptions that study participants are being exploited. Engaging the community can facilitate their understanding of the difference between research procedures and therapy [20]. The prospect for therapeutic misconception is also high and so research literacy efforts are required to prevent erroneous beliefs that instances of deaths or recovery during trials is necessarily the result of the experimental therapy. It is important that research teams engage with existing community structures to promote research literacy efforts by enhancing public discussions and understanding of national EVD research plans.

Formal community engagement can be achieved through the formation of community advisory boards (CABs). A CAB is composed of community members who share a common health concerns, history, symbols and language, and culture [21]. Their involvement in research may greatly improve the study design, informed consent process, and study implementation, particularly those targeting vulnerable populations. A community with low research literacy such as those in regions affected by EVD is considered vulnerable to research exploitation, making community engagement in EVD research more crucial.

It is important to acknowledge that in the wake of the EVD outbreak, communities have also responded by re-organizing themselves to ensure their protection and survival. For example, in the EVD affected regions, EVD survivors are organizing themselves into groups with the aim of addressing their particular needs such as addressing post-traumatic stress disorder, stigma, nutritional and housing needs; as well as engaging with the EVD response in their country [22]. EVD survivors would be excellent resource persons to work with during the design and implementation of EVD research.

While the engagement of representatives of communities and CABs may facilitate the perceived legitimacy of research, other factors need to be considered in forming ‘representative’ bodies for consultations. For example, it may be necessary also to engage stakeholders with expertise in infectious disease epidemics and who are familiar with the difficulties of undertaking research in such circumstances. This suggests that the membership of the CAB could include key stakeholders in the EVD response, those with higher research literacy and wider exposure to research (academicians, health practitioners, literate community members), and representatives of NGOs working in the area alongside representatives from the participant’s population such as EVD survivors.

The effectiveness of CABs within HIV research demonstrates the benefits of engaging communities with drug research and development, as articulated by West Slevin et al. [23]. With HIV treatment trials, CABs were composed of AIDS activists many of whom needed effective treatment to live. They were invested in promoting drug development for the management of HIV infection. This resulted in expedited review and approval of HIV drugs in the early 2000s. With HIV prevention research, CABs have been composed of more diverse people and have not been able to mobilise for stakeholder investment as effectively as it happened with HIV treatment activists [23]. With EVD research, it is advisable that the CABs should be composed of community members who feel ‘ strongly’ about the need to be directly engaged with the research and drug development process. This can lead to more significant impacts with the research and drug development process as was witness with the HIV treatment movement.

### Engagement of the ethics review committee

Conducting meaningful community engagement for EVD research during the current epidemic may be challenging in view of the urgent need to develop some forms of therapy that can help reduce case fatality. This scenario may promote the view that there is little time for extensive community consultative processes prior to commencement of research, unlike what happens for HIV prevention research. In the face of the current urgency, Folayan et al’s [24] proposal for community engagement for genomic research may be adopted as an alternative mechanism: efforts can be invested to indirectly engage communities in the design and implementation of EVD research through the engagement of laypersons on ethics committees. Laypersons are a ‘specialized’ subset of the community who actively participate in the administration of research protocols through their work on ethics committees. They can review protocols and provide constructive feedback that address community needs and concerns. This approach would require building the capacity of laypersons to review research protocols and address community concerns [25]. Where such capacity does not exist, this interim measure may not be a viable option.

### Stakeholder dialogue

It is important to ensure that stakeholder engagement is not done in silos. There is the need for effective communication mechanisms between different actors and players involved with the EVD response within countries affected by the outbreak, between countries in the EVD region and with other international stakeholders. Effective stakeholder engagement and dialogue can be facilitated using multiple media (tele-conferences, webinars, online dialogue) to promote sharing of best practices within and between affected countries and regions [26, 27]. Face to face community dialogue is equally important for facilitating discussions on EVD research in local communities [28, 29]. The current armamentarium of tools to facilitate intra- and inter- stakeholder communications can be harnessed to promote timely engagement between relevant stakeholders engaged with the EVD response.

## Summary

A coordinated local, regional and international response to the epidemic is essential. While there is a sense of urgency to develop therapies for the current EVD epidemic and to marshal available resources for the conduct of research on EVD [30–32], coordinated efforts should take place to ensure that international and local rules, laws and norms governing research are respected, exploitation of research participants is avoided and the EVD research process does not negatively impact upon patients and their support systems. This is important in view of the history of mistrust as the aftermath of civil wars in the West African region [33]. The collaboration between all stakeholders is crucial for continued long term partnership to address EVD outbreak and no one stakeholder should be left behind in ongoing efforts to develop EVD therapies.

## Abbreviation

EVD: Ebola Virus Disease
WHO: World Health Organisation
